# Adaptable Optical Fiber Displacement-Curvature Sensor Based on a Modal Michelson Interferometer with a Tapered Single Mode Fiber

**DOI:** 10.3390/s17061259

**Published:** 2017-06-02

**Authors:** G. Salceda-Delgado, A. Martinez-Rios, R. Selvas-Aguilar, R. I. Álvarez-Tamayo, A. Castillo-Guzman, B. Ibarra-Escamilla, V. M. Durán-Ramírez, L. F. Enriquez-Gomez

**Affiliations:** 1 Universidad Autónoma de Nuevo León, Pedro de Alba S/N, Ciudad Universitaria, 66455 San Nicolás de los Garza, Nuevo León, Mexico; romeo.selvasag@uanl.edu.mx (R.S.-A.); arturo.castillogz@uanl.edu.com (A.C.-G.); 2 Centro de Investigaciones en Óptica A. C., Loma del bosque 115, Col. Lomas del Campestre, 37150 León, Gto., Mexico; amr6@cio.mx (A.M.-R.); lfenriquez.gomez@gmail.com (L.F.E.-G.); 3 CONACYT – Universidad Autónoma de Nuevo León, Facultad de Ciencias Físico-Matemáticas, 66455 San Nicolás de los Garza, Nuevo León, Mexico; rialvarez@conacyt.mx; 4 Instituto Nacional de Astrofísica, Óptica y Electrónica (INAOE), L. E. Erro 1, Sta. Ma. Tonantzintla, Puebla 72824, Mexico; baldemar@inaoep.mx; 5 Centro Universitario de los Lagos, Universidad de Guadalajara, Enrique Díaz de León 1144, paseos de la montaña, 47460 Lagos de Moreno, Jalisco, Mexico; victor_duran@culagos.udg.mx; 6 Instituto Tecnológico de Aguascalientes, Avenida Adolfo López Mateos 1801, 20256 Aguascalientes, Ags., Mexico

**Keywords:** fiber optic sensor, modal optical fiber Michelson interferometer, displacement-curvature measurement

## Abstract

A compact, highly sensitive optical fiber displacement and curvature radius sensor is presented. The device consists of an adiabatic bi-conical fused fiber taper spliced to a single-mode fiber (SMF) segment with a flat face end. The bi-conical taper structure acts as a modal coupling device between core and cladding modes for the SMF segment. When the bi-conical taper is bent by an axial displacement, the symmetrical bi-conical shape of the tapered structure is stressed, causing a change in the refractive index profile which becomes asymmetric. As a result, the taper adiabaticity is lost, and interference between modes appears. As the bending increases, a small change in the fringe visibility and a wavelength shift on the periodical reflection spectrum of the in-fiber interferometer is produced. The displacement sensitivity and the spectral periodicity of the device can be adjusted by the proper selection of the SMF length. Sensitivities from around 1.93 to 3.4 nm/mm were obtained for SMF length between 7.5 and 12.5 cm. Both sensor interrogations, wavelength shift and visibility contrast, can be used to measure displacement and curvature radius magnitudes.

## 1. Introduction

Optical fiber sensors applied to the measurement of physical, chemical, and biological parameters have been a subject of great interest since they have special advantages such as simplicity, compactness, immunity to electromagnetic field, easy construction, fast response, resistance to hazard environments, and real time measurement, among others. Moreover, mechanical bending is an important physical parameter to be measured in order to determine deformations, displacements, and curvature radius for high resolution optical instrumentation processes. In recent years, several bending sensors based on optical fiber structures have been reported [1,2,3,4,5,6,7,8,9,10]. Most of them are based on the modal Mach-Zehnder interferometer (MZI), whose construction is by means of optical fiber devices which couples light propagation modes between core and cladding, such as tapered fibers [8], long-period fiber gratings (LPFG) [9], fiber Bragg gratings (FBG) [10], and micro-fiber lenses [3], among others. In the aforementioned in-fiber structures, the use of two mode coupler devices is necessary to construct an MZI, however, the fabrication process for FBGs, LPFGs, and spliced spherical shape structures is not quite as simple. In the case of fiber tapers, also by means of just one taper, an MZI can be constructed [11], but the taper needs to be abrupt, increasing the thinness and fragility of the tapered fiber. Furthermore, the use of special fibers to generate both arms of the MZI has been used for bending measurement, e.g., multicore fiber [2,12] and photonic crystal fiber [13]. Nevertheless, the structure becomes more complex and unrepeatable due to the splice collapses, as well as more expensive because of the use of special fibers that are not readily accessible. A simpler structure than the Mach-Zehnder interferometer is the optical fiber Michelson interferometer (OFMI), in which only one optical fiber mode coupler device is required to construct the interferometer. The OFMI as a fiber sensing device has been reported to measure refractive index [14], pressure [15], temperature [16], and strain [17] by using fiber structures based on tapered fiber, photonic crystal fiber, multicore fiber, and large core diameter fiber, respectively.

In this work, we propose and experimentally demonstrate a robust, simple, and adjustable sensor for mechanical axial displacement or bending measurements based on an OFMI structure. The Michelson interferometer is constructed by a single-mode fiber (SMF) adiabatic bi-conical taper spliced to an SMF segment. The core and cladding of the SMF segment act as the two paths of the Michelson interferometer. When the bi-conical taper is straight, no interference between modes is presented. In contrast, when the taper is bent, an interference pattern is produced by modal interference which, with the interferometric structure, corresponds to an OFMI spectrum. By increasing the bending of the bi-conical taper with an axial displacement, a wavelength spectral shift and a small fringe contrast in the notches of the interference pattern of the OFMI reflection spectrum are produced. Taking advantage of these characteristics, we construct an optical fiber displacement-curvature sensor. As a special sensor characteristic, both the magnitude value of the wavelength shift and the spectral shape of the device depend on the SMF segment length, which can be tailored. Sensitivities from around 1.93 to 3.4 nm/mm and a fringe period spectrum ranging from ~6.21 to ~1.8 nm were experimentally obtained for SMF lengths from 7.5 cm to 15 cm.

## 2. Structure and Working Principle of the Proposed Optical Fiber Michelson Interferometer (OFMI)

**Figure 1:**
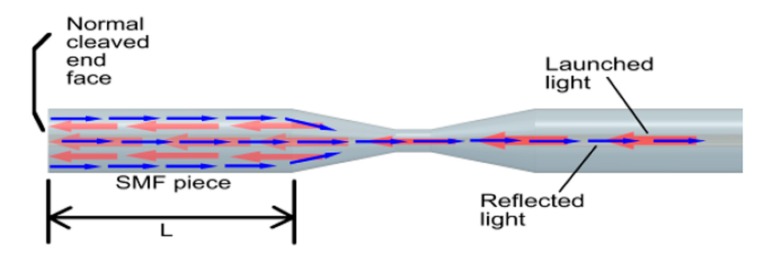
Structure of the modal Michelson interferometer with the light modes propagation.

The structure of the proposed OFMI and the light modes propagation through the device are shown in Figure 1. An optical fiber taper spliced to an SMF segment with length L comprise the sensing device. The light that is launched to the core fiber at the input is propagated through the tapered fiber (left pointing red arrows). In the taper position, the fundamental mode of the core fiber couples part of its energy to cladding modes. Then, both modes from the core and cladding travel through the SMF segment. In the normal cleaved end face of the SMF, the ~4% energy modes are reflected by Fresnel reflection (right pointing blue arrows) and propagate in opposite directions through the taper, where the cladding mode recouples part of the energy to the core mode. 

Because the core and cladding modes have different propagation constants; an interference pattern is produced from the reflected light. The generated phase difference, which causes the mode interference between core and cladding modes, can be approximated by the expression [14]:

(1) $$\varphi =\frac{4\pi \Delta n_{eff}L}{\lambda }$$

where $\Delta n_{eff}$ is the difference of effective refractive index of core and cladding modes, *L* is the optical path length, and *λ* is the input wavelength in vacuum.

**Figure 2:**
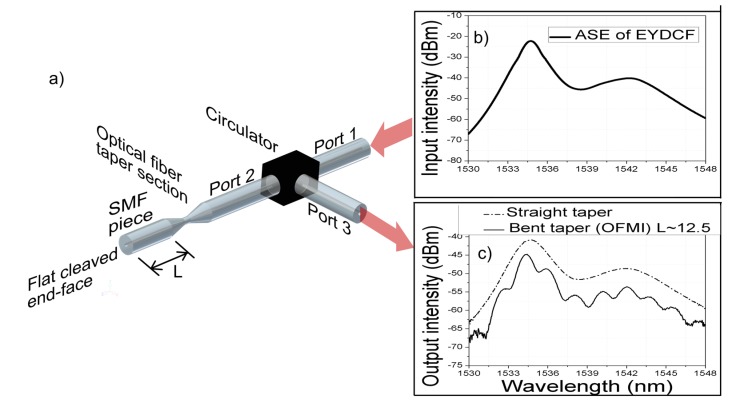
(**a**) Schematic of the OFMI measurement; (**b**) input spectrum intensity from an EYDCF to circulator port 1; (**c**) Output spectrum intensity from circulator port 3 to an OSA for measurement.

The tapered optical fiber is adiabatic and has a bi-conical shape with transition lengths of 2.5 mm, a waist length of 1 mm, and a waist diameter of 60 µm. The fiber was tapered with the mentioned dimensions by using a glass processing machine Vytran model GPX-3400 manufactured by Vytran LLC at Morganville, NJ, USA. As the dimensions of a bi-conical fused taper are essential to its functioning as an interferometric device [18], the taper dimensions were selected in order to have a taper that is adiabatic, yet at the limit in which the taper gradually loses its adiabaticity by a slight deformation such as bending. Considering other taper dimensions, such as waist size, waist length, and transitions length, will result in differences in the adiabaticity and spectral properties of the OFMI. Using a fiber circulator, the reflected output spectrum of the OFMI from the amplified spontaneous emission (ASE) of an Erbium-Ytterbium double cladding fiber (EYDCF) was measured by an Optical Spectrum Analyzer (OSA) with a resolution of 0.07 nm, as seen in Figure 2. The spectra of the ASE input signal and the reflected output signal of the OFMI when the mode coupler is straight and when it is bent to a radius of ~48,208.75 mm is also shown in Figure 2. As can be observed, while the tapered optical fiber is fixed in a straight position, the output spectrum is just the ASE reflected light from the SMF segment, since the taper is not performing as a mode coupler until it is perturbed by bending.

**Figure 3:**
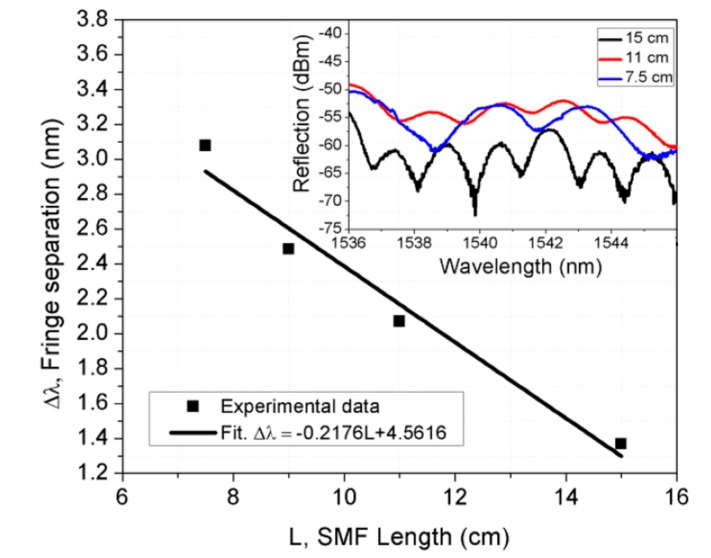
Interference fringe separation of the Michelson interference pattern versus length of single mode fiber piece. The inset shows the spectra for OFMIs with SMF lengths of 7.5, 11, and 15 cm.

The wavelength period of the OFMI as a function of the SMF length is depicted in Figure 3. As can be observed, the wavelength period decreases as *L* increases. The experimental results can be approximately fitted to:

(2) Δλ=−0.2176L+4.5616

where Δ*λ* denotes the wavelength fringe separation as a function of the length of the SMF piece. For the proposed OFMI-based sensor, the SMF length range varied from 7.5 to 15 cm, where the data can be approximated to a linear fit maximum slope value of around −0.2176 nm/cm. The inset in Figure 3 shows the measured reflected interference pattern spectra for the bent OFMI with 15, 11, and 7.5 cm of *L* when they are bent to a curvature radius ~48,208 mm.

## 3. Analysis and Experimental Results

**Figure 4:**
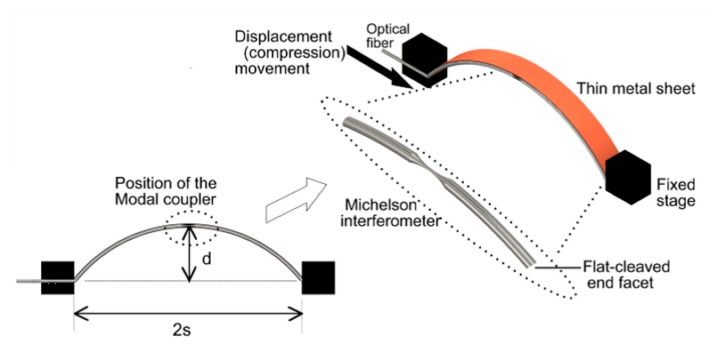
Schematic of the mechanical stage for the displacement and curvature radius characterization of the Michelson interferometer.

The schematic of the stage used to characterize the OFMI by mechanical displacement or bending is shown in Figure 4. The OFMI was mounted in a fabricated V groove in a flexible metal sheet ensuring that the bi-conical taper was positioned in the middle of the sheet whose total length was 29.1 cm. The OFMI was glued only on one metal sheet end to avoid the fiber being longitudinally stressed when bending and prevent the fiber from breaking. The metal sheet was compressed by the axial displacement of a micrometric linear translation stage. The corresponding bending for every displacement position was calculated by using the formula [12]:

(3) $$r=\frac{d^{2}+s^{2}}{2d}$$

where 2*s* is the distance between the two ends of the metal sheet, and *d* is the maximum deflection of the metal sheet (see Figure 4).

**Figure 5:**
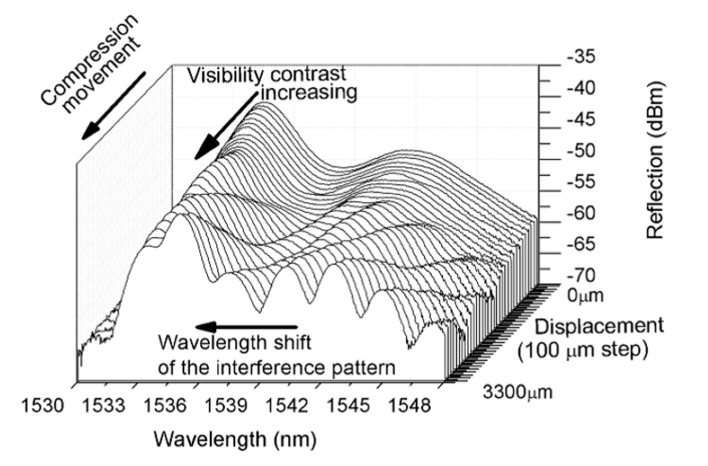
OFMI output spectra for several mechanical displacement positions separated by 100 µm for a device with *L* ~ 10 cm.

**Figure 6:**
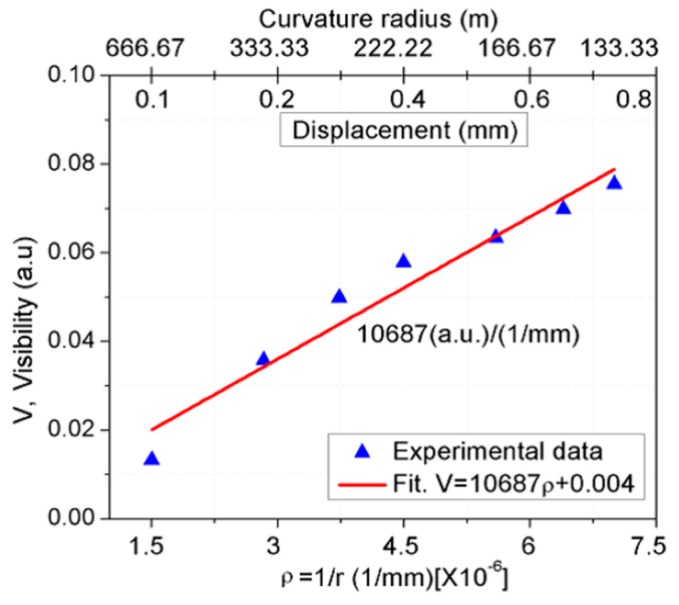
Spectral visibility response for a device with 7.5 mm length of SMF.

When the linear translation stage compresses the metal sheet, the device is bent and its curvature radius decreases. As a result, the spectral response of the OFMI shifts toward shorter wavelengths and a small visibility increase is also caused in the interference pattern of the spectrum, as can be observed in Figure 5, from zero displacement to 3300 µm of a device with ~10 cm of SMF length, which are the straight position and a curvature radius of 55,448.53 mm, respectively. In Figure 5, the upper curve is the measurement output spectrum when the device is straight, i.e., without modal interference. As the displacement is applied, the device is bent, modal interference takes place, and the fringe contrast of the notches in the interference pattern increases as the displacement increases while a wavelength shift is also caused. Figure 6 shows the spectral visibility sensor response for a device with 7.5 mm of SMF segment length. An increase in visibility was observed as the inverse curvature radius (ρ) increases from 1.5 × 10^−6^ to 7 × 10^−6^ mm^−1^ with an approximate linear factor of around 10,687 (a.u.)/(1/mm). Different sensor lengths were tested and they show similar visibility increase.

**Figure 7:**
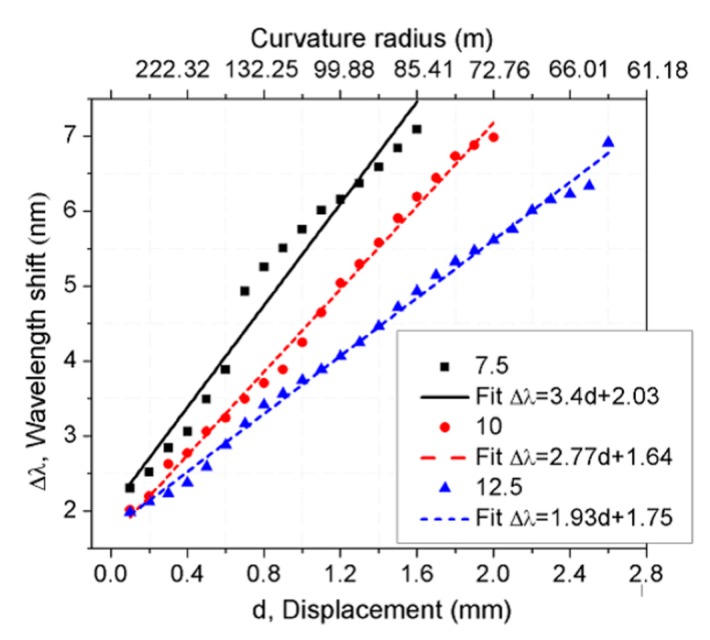
Comparison of the wavelength shift response caused by mechanical displacement and bending for devices with 7.5, 10, and 12.5 cm length values of SMF lengths segments.

For L values of 7.5, 10, and 12.5 cm, the wavelength shift of the OFMI output spectrum as a function of the mechanical displacement and curvature radius (m) is shown in Figure 7. The corresponding curvature radius for each displacement position is indicated at the top of the graphic. The response of the three different SMF lengths exhibits an approximately linear behavior. The linear fitting of the experimental data can be fitted by the following equations:

(4) $$\begin{array}{l} \Delta \lambda_{L=7.5}=3.4d+2.03\\ \Delta \lambda_{L=10}=2.77d+1.64\\ \Delta \lambda_{L=12.5}=1.93d+1.75 \end{array}$$

where Δ*λ* is the wavelength shift of the OFMI output spectrum and *d* is the value of the mechanical axial displacement of the linear translation stage. As can be observed, the slope increases from 1.93 to 3.4 nm/mm as the *L* value decreases.

**Figure 8:**
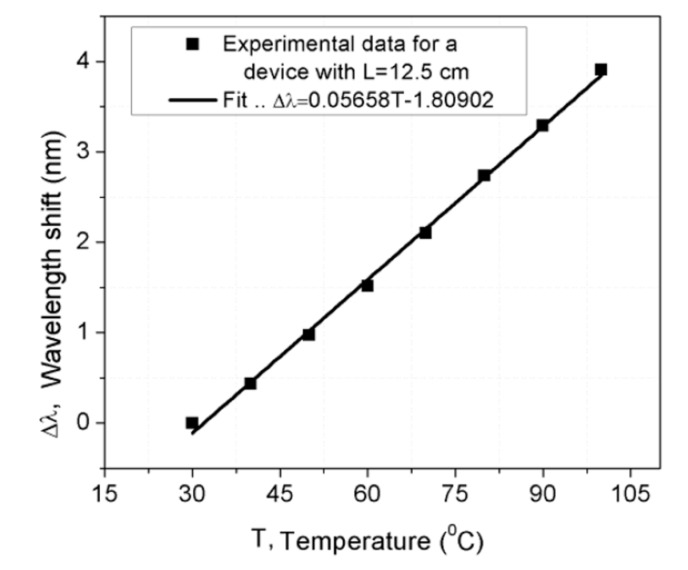
Wavelength shift versus temperature of an OFMI with an SMF length of 12.5 mm.

As the effect of temperature is an important parameter to be considered in sensor performance, the OFMI was exposed to temperature changes. An OFMI with an SMF length of 12.5 mm was positioned on a hot plate when it was bent to a bending radius of around 48,208 mm, and a spectral shift of 3.7 nm to larger wavelengths with negligible visibility change was observed as the temperature increases from room temperature to 100 °C. Figure 8 shows the wavelength shift versus the temperature of this OFMI with a length of 12.5 mm; the wavelength shift increases in a linear way with a factor of ~0.05658 nm/°C. As the OFMI is sensible to temperature changes, temperature perturbations can alter the sensor bending measurement. However, this can be avoided by maintaining the sensor in a temperature-controlled environment.

A special characteristic to add to our sensor is that the straight position of the sensor can be designed as the zero initial condition; for this case, the output is the reflected spectrum without modal interference (see upper spectrum in Figure 5). The tapering fabrication for the construction of the sensor is simple, fast, and repeatable. The manufacturing process for the sensor construction simply employs a splicer machine to splice standard fiber to a bi-conical tapered fiber. 

When the sensor is used to measure the displacement related to bending, the spectral wavelength shift sensor response can be adjusted by varying the length of an SMF, which can be done by simply cutting or splicing standard fiber. Also, the spectral shape response of the sensor can be modified by changing the SMF length, and the sensor interrogation can be done by either wavelength shift or spectral visibility change. These characteristics are very useful to adapt the sensor to special conditions for a particular measurement application.

## 4. Conclusions

In conclusion, a robust, simple, and highly sensitive optical fiber mechanical axial displacement or curvature radius sensor based on a Michelson interferometer using an adiabatic bi-conical taper, which can be interrogated by spectral wavelength shift or visibility change, has been demonstrated for the first time to our knowledge. The tapered optical fiber, which is compact, is spliced with an SMF segment to conform the sensor. When the sensor is bent by applying an axial displacement, the symmetry of the taper is modified, which causes a variation in the taper refractive index profile and a breaking down of the tapered adiabaticity to couple modes and interference between them. As a consequence of such a perturbation, a phase delay between modes causes a wavelength shift of the output spectrum device to smaller wavelength values as well as an increase of visibility in the fringes of the sensor interference pattern, due to the loss of the tapered adiabaticity which enables mode coupling. As an additional advantage, for the need of a specific application, the mechanical axial displacement sensitivity by wavelength shift and the fringe separation sensor spectrum can be tailored or adjusted by changing the length of the single-mode segment spliced to the taper by simple splicing or cutting an SMF. The proposed optical fiber displacement sensor based on a Michelson interferometer is shown to be a reliable optical fiber device for high resolution optical instrumentation applications.
